# Dietary flavonoid intake is linked to reduced all-cause mortality: Findings from the National Health and Nutrition Examination Survey (NHANES)

**DOI:** 10.12669/pjms.42.2.13618

**Published:** 2026-02

**Authors:** Jinhua Wei, Yuting Wang

**Affiliations:** 1 Jinhua Wei Department of Gynecology, Zhongshan Hospital (Xiamen), Fudan University, Xiamen, Fujian Province 361015, P.R. China; 2 Yuting Wang Department of Intensive Care Unit, Zhongshan Hospital (Xiamen), Fudan University, Xiamen, Fujian Province 361015, P.R. China

**Keywords:** Flavonoids intake, All-cause mortality, U-shaped association, NHANES

## Abstract

**Objective:**

To examine the relationship between dietary flavonoid consumption and all-cause mortality (ACM) among U.S. adults, and determine the optimal intake levels and target populations that could benefit from such dietary interventions.

**Methodology:**

A cohort study employing data from the National Health and Nutrition Examination Survey (NHANES) was used with follow-up for ACM status until December 31, 2018. This was a United States population-based study employing NHANES data. A total of 4,60 adults from NHANES were asked about their dietary flavonoid intake via 24 hours dietary recall.

**Results:**

After a median follow-up of 121 months, 51 mortality cases were recorded. Analysis found a U-shaped correlation between dietary flavonoid consumption and ACM, with optimal intake levels ranging from 48.93 mg to 735.65 mg. Subgroup analysis revealed that males (P = 0.024), smokers (P = 0.010), non-drinkers (P = 0.025), and individuals without obesity (P = 0.003) and cardiovascular disease (P = 0.008) benefited the most from the dietary flavonoid intake.

**Conclusions:**

Optimal dietary flavonoid intake is linked to reduced ACM, especially in males, smokers, non-drinkers, and individuals without obesity or cardiovascular disease. These findings show that personalized dietary recommendations may be important for improving health outcomes.

## INTRODUCTION

Dietary flavonoids are a wide range of polyphenolic bioactive compounds found in fruits, vegetables, wine, and other plant-based foods that are consumed daily.^1^,^2^ Recent studies have showed that flavonoids can have antioxidant, anti-inflammatory, and cardioprotective effects, and can reduce overall mortality.^2^-^5^ However, the exact association of flavonoids with all-cause mortality (ACM) is still unclear. Some epidemiological studies have confirmed a significant negative correlation between the intake of flavonoids and the incidence of ACM, indicating that consuming more flavonoids may reduce the risk of mortality.^5^,^6^ In contrast, Hooper et al.^7^ found no link between flavonoid intake and ACM. Such a discrepancy may be due to differences in research design, participant demographics, flavonoid consumption levels, and dietary assessments.^6^-^8^ In addition, the different biological activities of specific subclasses of flavonoids (such as flavanols, flavanones, flavonoids, and anthocyanins) may lead to heterogeneous health effects, making it more difficult to explain the overall research results.^9^ Therefore, there is a pressing need to clarify the potential association between dietary exposure and ACM risk in different populations through the use of comprehensive dietary assessments and advanced statistical methods.^10^

The National Center for Health Statistics of the Centers for Disease Control and Prevention in the United States established the National Health and Nutrition Examination Survey (NHANES), which contains a large amount of systematically collected information on the dietary intake and health-related outcomes of a diverse population of American adults. This comprehensive and authoritative data has become an essential resource for analyzing the association between flavonoid intake and ACM.

Unlike previous cohort studies conducted predominantly in homogeneous European populations,^2^,^4^ this study utilized data from NHANES—a nationally representative U.S. survey characterized by diversity in race/ethnicity, socioeconomic status, and dietary behaviors. In addition, this study is the first, to our knowledge, to quantify an optimal flavonoid intake range (48.93–735.65 mg/day) associated with the lowest all-cause mortality using restricted cubic spline modeling. Furthermore, comprehensive subgroup analyses allowed us to identify population subgroups (males, smokers, non-obese individuals) who may derive the greatest benefit, thus enhancing the translational relevance of our findings.

This study aimed to use NHANES data to analyze the relationship between dietary flavonoid intake and ACM, and to clarify the optimal intake of flavonoids that can reduce the risk of ACM. The study may allow for identifying specific populations that may benefit the most from increased consumption of flavonoids. The results of this study may provide valuable insights into public health recommendations and dietary guidelines that aim to promote longevity and reduce the burden of chronic diseases.

## METHODOLOGY

NHANES data is released every two years. This study used NHANES cycle data from 1999 to 2018, and mortality data as of December 31, 2018. As all data is fully public, no ethical approval was required.

### Exclusion criteria:

- Flavonoid dietary intake data missing.
- Anthropometric measurements not available:
- Physical examinations or biochemical analyses data incomplete.
- Socio-demographic, lifestyle, and medical records not fully documented.

### Study Population and Data Processing:

**Fig.1:**
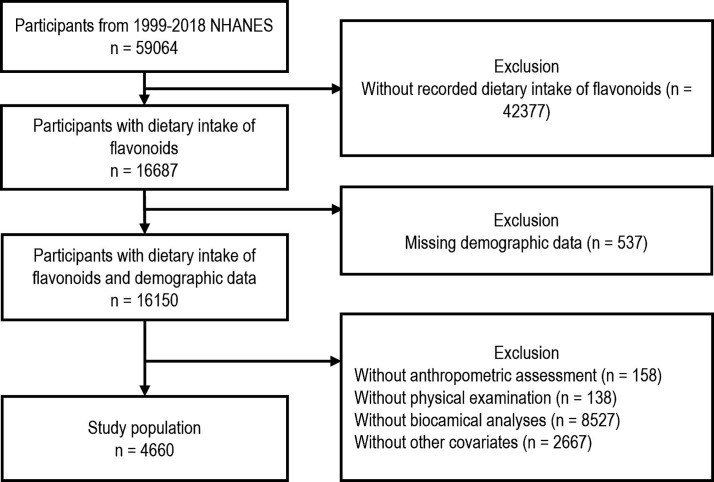
Flowchart illustrating the Inclusion and Exclusion Criteria for the Study Population.

This study employed a complete-case analysis approach. Participants with missing data in any key domain—including flavonoid intake, demographic variables, anthropometric measurements, physical examination, biochemical testing, and covariates—were excluded. As illustrated in Fig. 1, the initial NHANES sample of 59,064 individuals was sequentially reduced based on data availability, resulting in a final analytic sample of 4,660. Due to extensive missingness in flavonoid intake for some participants, multiple imputation was not performed. Moreover, the modular design of NHANES, rather than selective attrition, was the primary source of missingness, making listwise deletion a methodologically acceptable strategy.

### Dietary assessment:

Flavonoid intake data were gathered via the U.S. Department of Agriculture (USDA) Automated Multiple-Pass Method (AMPM) using a single 24 hours dietary recall. Although a second-day recall is available in some NHANES cycles, we used only the first-day recall to maintain consistency across survey years and participants. Foods were coded using the USDA Food and Nutrient Database for Dietary Studies (FNDS) and linked to flavonoid values from the USDA Flavonoid Database for Survey Food Codes (207–2010 and 2017–2018). This database categorizes flavonoids into six subclasses: anthocyanidins, flavan-3-ols, flavanones, flavanols, and isoflavones, which cover 29 distinct types. Total flavonoid intake is defined as the sum of the average values from these subclasses. No energy adjustment was applied, as our analysis focused on absolute intake levels.

### Outcomes:

The primary outcome is all-cause mortality, defined as death from any cause through December 31, 2018. The mortality data come from 19-204 mortality records related to the NHANES public use. This study analyzed the period from enrollment to death or review, marked by interview dates. During the entire follow-up period, participants who did not match any death records were considered alive.

### Variables:

This study conducted statistical analyses on a series of variables, including anthropometric assessment, physical examination, biochemical testing, and demographic information. Social and economic factors, such as age, gender, education level, family income poverty rate (PIR), lifestyle behaviors such as smoking and drinking, and medical history of diabetes, hypertension, and hyperlipidemia were collected through individual interviews. Hypertension was defined as the blood pressure reaching 140/90 milligrams or higher, which is a pre-diagnosis or the use of antihypertensive drugs. Hyperlipidemia was determined by total cholesterol levels exceeding 5.0 mol/L, low-density lipoprotein (LDL) cholesterol levels reaching 3 mol/L or higher, triglyceride levels exceeding 1.7 mol/L, or the use of lipid-lowering drugs. Diabetes was defined as previously diagnosed, the fasting blood glucose level of 7 mol/L or higher, or the use of anti-diabetes drugs. Body Mass Index (BMI) = weight (kg) / height (m^2^).

Blood pressure measurements were conducted employing a mercury sphygmomanometer, following the American Heart Association’s guidelines for sphygmomanometer-based blood pressure measurement. Biochemical tests such as glucose, total cholesterol, triglycerides, creatine, and urea nitrogen were performed using a Hitachi Model 704 multi-channel analyzer (Boehringer Mannheim Diagnostics, Indianapolis, Indiana). The level of hemoglobin A1c was measured by an automated glycated hemoglobin analyzer using high-performance liquid chromatography (HPLC) (Primus Corporation). The direct immunoassay method was used to measure high-density lipoprotein (HDL) cholesterol, and the Friedel Ewald equation was used to calculate LDL cholesterol levels. Measurement of high-sensitivity C-reactive protein (Hs CRP) using the latex-enhanced scattering turbidity method.

### Statistical analysis:

The study participants’ characteristics were summarized using percentages (%) or medians, along with interquartile ranges (IQR). The normality of continuous variables was evaluated via the Kolmogorov-Smirnov test. For non-normally distributed variables, comparisons between groups were conducted using the Mann-Whitney U test, whereas for categorical variables, the chi-square (χ^2^) test was used. Spearman’s rank correlation was used to assess the relationships between dietary flavonoid intake and cardiovascular risk factors, since Spearman’s method is appropriate for both continuous and ordinal variables, and doesn’t require linearity or normal distribution assumptions. Multivariable Cox regression models were employed to evaluate the association between dietary flavonoid consumption and ACM, while accounting for a variety of potential confounders, including age, gender, PIR (Pepcid), educational attainment, smoking habits, alcohol intake, BMI, waist circumference, blood pressure (systolic and diastolic), diabetes, hypertension, hyperlipidemia, cardiovascular disease, and medication use. Restricted cubic spline regression was used to investigate potential nonlinear correlations between flavonoid intake and acidemia, enabling a more flexible modeling approach for the dose-response relationship. Subgroup analyses were carried out with stratified Cox regression models to determine populations that might gain the most benefit from optimal flavonoid intake. All statistical analyses were performed using R software (version 4.2.2), and a P-value < 0.05 was considered statistically significant.

## RESULTS

Over a median follow-up period of 121 months, 51 deaths were recorded among 460 participants in the analysis. Fig.1 shows the screening process for participants. Table-I shows the baseline characteristics of the study participants, divided into two groups: one group represents survivors, the other represents deaths. Table-I. Participants in the death group were significantly older compared to those in the survival group (median age: 74.00 vs. 47.00 years, P < 0.001), and had higher proportions of males, lower educational attainment, and lower income levels. Additionally, the death group exhibited significantly higher rates of diabetes, hypertension, and hyperlipidemia (P < 0.001 for all).

**Table-I:** Baseline Characteristics of Study Participants.

Characteristics median (IQR) or %	Total (N=4660)	Survival group (N=4149)	Death group (N=511)	Z/χ^2^	P
Age, year	50.00 [35.00, 64.00]	47.00 [33.00, 61.00]	74.00 [64.00, 80.00]	-27.259	<0.001
Gender				12.599	<0.001
Female	2315 (49.7)	2099 (50.6)	216 (42.3)		
Male	2345 (50.3)	2050 (49.4)	295 (57.7)		
High educational level	2390 (51.3)	2224 (53.6)	166 (32.5)	81.214	<0.001
PIR	2.19 [1.18, 4.25]	2.30 [1.20, 4.37]	1.75 [1.08, 2.93]	-5.763	<0.001
Use of tobacco	2077 (44.6)	1775 (42.8)	302 (59.1)	49.039	<0.001
Use of alcohol	4081 (87.6)	3653 (88.0)	428 (83.8)	7.688	0.007
BMI, kg/m^2^	28.00 [24.50, 32.39]	28.02 [24.55, 32.50]	27.51 [24.10, 31.64]	-2.182	0.029
Waist circumference, cm	98.20 [88.00, 108.70]	97.80 [87.50, 108.30]	100.80 [91.75, 111.20]	-4.909	<0.001
Diabetes mellitus	1809 (38.8)	1469 (35.4)	340 (66.5)	185.641	<0.001
Hypertension	1954 (41.9)	1587 (38.3)	367 (71.8)	210.569	<0.001
Hyperlipidemia	3398 (72.9)	2969 (71.6)	429 (84.0)	35.389	<0.001
Cardiovascular disease	510 (10.9)	323 (7.8)	187 (36.6)	387.450	<0.001
Use of antidiabetic drugs	523 (11.2)	400 (9.6)	123 (24.1)	95.076	<0.001
Use of antihypertensive drugs	1504 (32.3)	1158 (27.9)	346 (67.7)	329.710	<0.001
Use of lipid lowering drugs	952 (20.4)	731 (17.6)	221 (43.2)	183.851	<0.001
Systolic pressure, mmHg	120.00 [110.00, 132.00]	119.00 [110.00, 130.00]	130.00 [117.00, 145.00]	-11.279	<0.001
Diastolic pressure, mmHg	70.00 [62.00, 77.00]	71.00 [63.00, 78.00]	65.00 [55.00, 74.00]	-9.486	<0.001
Glucose, mmol/L	5.22 [4.83, 5.77]	5.22 [4.83, 5.72]	5.61 [5.08, 6.44]	-10.457	<0.001
HbA1c, %	5.50 [5.20, 5.90]	5.50 [5.20, 5.80]	5.80 [5.50, 6.30]	-13.098	<0.001
Creatinine, umol/L	74.26 [63.65, 88.40]	72.49 [62.76, 85.75]	84.86 [72.49, 104.75]	-12.809	<0.001
Uric acid, umol_L	327.10 [267.70, 380.70]	321.20 [267.70, 374.70]	350.90 [291.50, 410.40]	-6.843	<0.001
Urea nitrogen, mmol/L	4.64 [3.57, 5.71]	4.28 [3.57, 5.71]	5.71 [3.93, 7.50]	-11.869	<0.001
Triglycerides, mmol/L	1.17 [0.84, 1.71]	1.17 [0.82, 1.69]	1.21 [0.89, 1.71]	-2.261	0.024
Total cholesterol, mmol/L	4.93 [4.27, 5.66]	4.94 [4.29, 5.66]	4.84 [4.11, 5.65]	-2.324	0.02
HDL-cholesterol, mmol/L	1.32 [1.11, 1.60]	1.32 [1.11, 1.60]	1.32 [1.09, 1.63]	-0.255	0.799
LDL-cholesterol, mmol/L	2.90 [2.35, 3.54]	2.92 [2.35, 3.54]	2.79 [2.16, 3.46]	-3.403	0.001
Hs-CRP, mg/L	1.80 [0.79, 4.10]	1.72 [0.73, 4.00]	2.20 [0.90, 5.05]	-11.500	<0.001
Total isoflavones, mg	0.00 [0.00, 0.05]	0.00 [0.00, 0.05]	0.00 [0.00, 0.02]	-5.557	<0.001
Total anthocyanidins, mg	0.76 [0.00, 7.44]	0.75 [0.00, 7.56]	0.86 [0.00, 6.35]	-0.866	0.386
Total flavan-3-ols, mg	10.78 [2.56, 63.55]	10.85 [2.55, 62.59]	10.45 [2.66, 83.16]	-0.323	0.747
Total flavanones, mg	0.23 [0.00, 6.05]	0.23 [0.00, 5.85]	0.20 [0.00, 7.39]	-0.908	0.364
Total flavones, mg	0.40 [0.08, 1.09]	0.42 [0.09, 1.12]	0.34 [0.06, 0.86]	-3.190	0.001
Total flavanols, mg	12.42 [5.80, 24.17]	12.59 [5.91, 24.56]	10.93 [4.85, 20.92]	-3.312	0.001
Total sum of all flavonoids, mg	48.93 [17.08, 181.80]	49.04 [17.33, 183.44]	47.76 [15.75, 170.10]	-0.977	0.329

PIR: income-to-poverty ratio; BMI: body mass index; HbA1c: hemoglobin A1c; HDL: high-density lipoprotein; LDL: low-density lipoprotein; Hs-CRP: high-sensitivity C-reactive protein

The linear trend analysis didn’t reveal a significant linear relationship between total flavonoid intake and ACM across different models. Table-II In Model-1 (unadjusted), the quartiles of flavonoid intake were not significantly linked to ACM, with hazard ratios (HRs) of 0.797, 0.931, and 0.875 for quartiles 2-4, respectively. However, after accounting for several covariates in Models two and three, a U-shaped relationship emerged. A significant reduction in mortality was observed for quartiles two and four compared to the reference group (P = 0.008 for Q2 in Model-2 and P = 0.027 for Q2 in Model-3), suggesting that moderate flavonoid intake is more beneficial than very low or very high intake.

**Table-II:** Linear trend analysis of the association between dietary flavonoid intake and all-cause mortality.

Variables	Model1		Model2		Model3	
	HR (95%CI)	P	HR (95%CI)	P	HR (95%CI)	P
Total sum of all flavonoids						
Q1	1.000 (Reference)		1.000 (Reference)		1.000 (Reference)	
Q2	0.797 (0.624 ~ 1.018)	0.069	0.713 (0.555 ~ 0.915)	0.008	0.753 (0.585 ~ 0.969)	0.027
Q3	0.931 (0.733 ~ 1.184)	0.561	0.859 (0.673 ~ 1.097)	0.223	0.887 (0.695 ~ 1.133)	0.336
Q4	0.875 (0.687 ~ 1.115)	0.282	0.757 (0.591 ~ 0.969)	0.027	0.810 (0.632 ~ 1.039)	0.098
HR: Hazard Ratio, CI: Confidence Interval, Q1: ≤17.08mg, Q2: >17.08mg and ≤48.93mg, Q3: >48.93mg and ≤181.83mg, Q4: >181.83mg						
Model1: Crude						
Model2: Adjusted for: age, gender, PIR, high educational level, use of tobacco, use of alcohol, BMI, waist circumference, systolic pressure, diastolic pressure, diabetes mellitus, hypertension, hyperlipidemia, cardiovascular disease, use of antidiabetic drugs, use of antihypertensive drugs, use of lipid-lowering drugs						
Model3: Adjusted for: age, gender, PIR, high educational level, use of tobacco, use of alcohol, BMI, waist circumference, systolic pressure, diastolic pressure, diabetes mellitus, hypertension, hyperlipidemia, cardiovascular disease, use of antidiabetic drugs, use of antihypertensive drugs, use of lipid lowering drugs, glucose, HbA1c, creatinine, uric acid, urea nitrogen, triglycerides, total cholesterol, HDL-cholesterol, LDL-cholesterol, Hs-CRP.						
HR: Hazard Ratio, CI: Confidence Interval						

**Fig.2:**
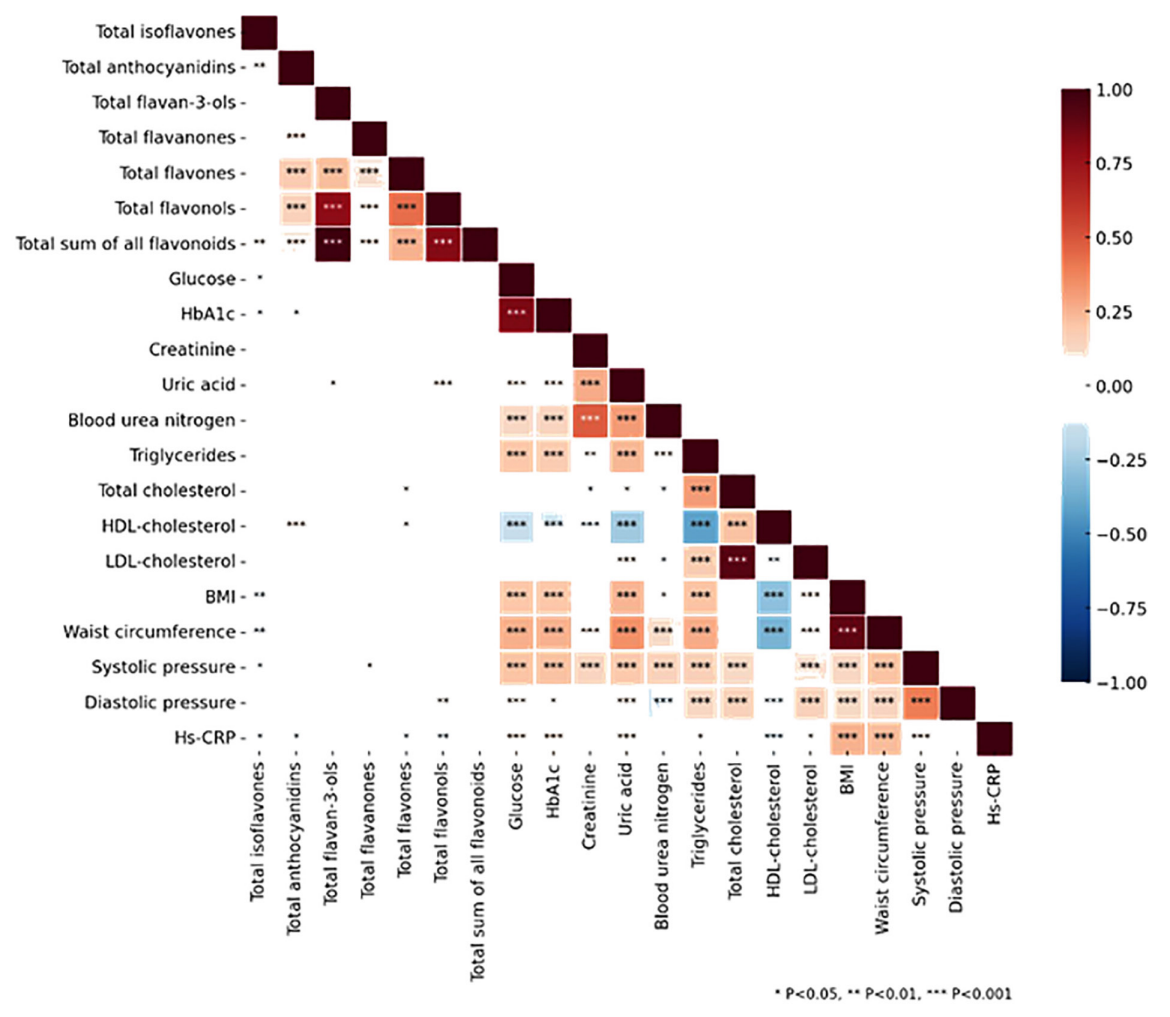
presents the correlation analysis between dietary flavonoid intake and cardiovascular risk factors.

The relationship between dietary flavonoid consumption and cardiovascular risk factors is shown in Fig-2. There was a significant inverse correlation between total flavonoid intake and key risk factors like glucose, HbA1c, and systolic blood pressure, suggesting potential cardioprotective effects of flavonoids. These associations show that flavonoids play a role in modulating cardiovascular health, which might affect overall mortality risk.

**Fig.3:**
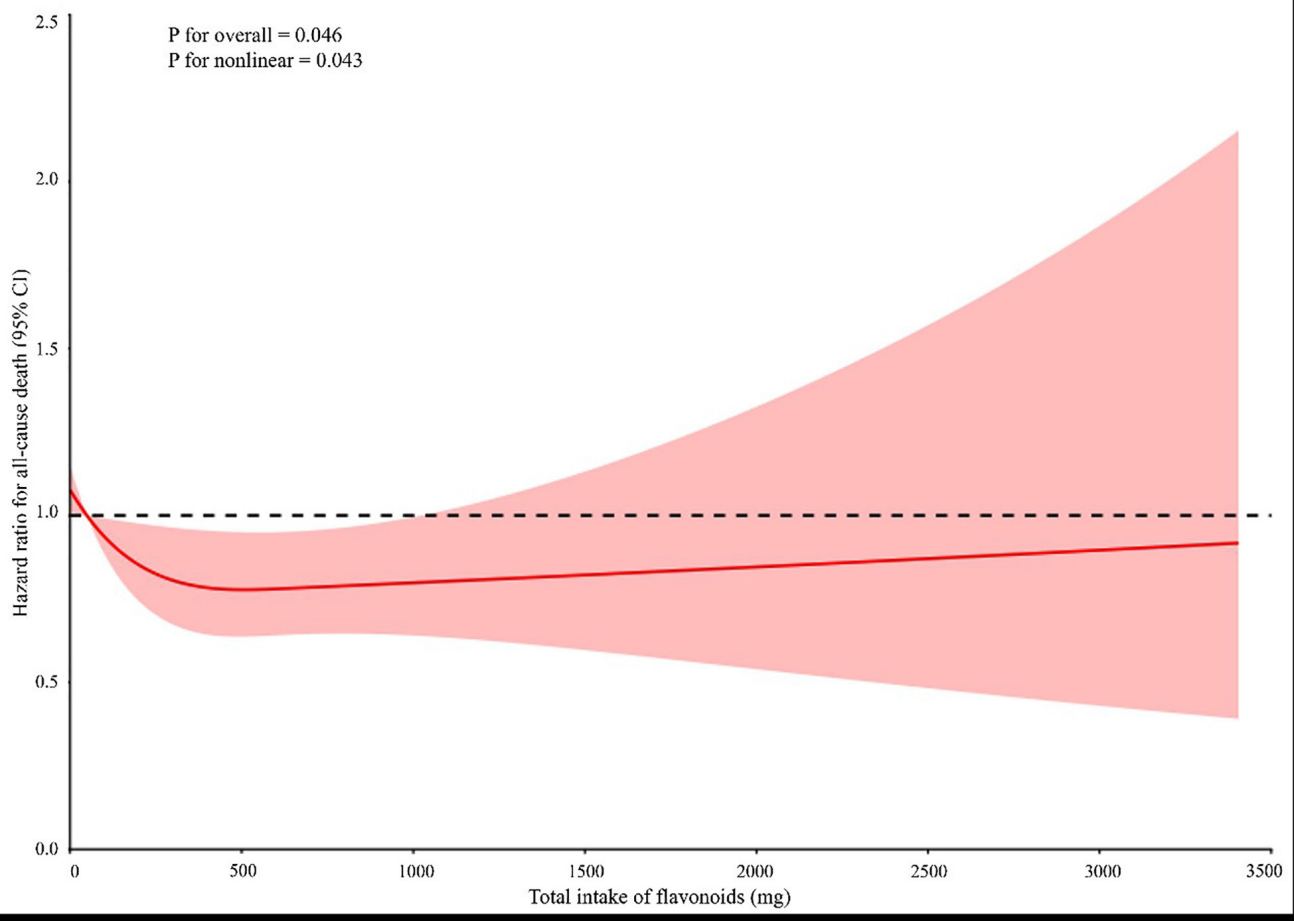
illustrates the association between dietary flavonoid intake and al-cause mortality. Inflection point: 139.58 mg; Optimal flavonoid intake (interval corresponding to lowest hazard ratio): 48.93 mg to 735.65 mg.

As shown in Fig.3, the restricted cubic spline analysis demonstrated the U-shaped correlation between dietary flavonoid consumption and all-cause mortality. The study found that the optimal intake range was 48.93 to 735.65 mg/day and was associated with the lowest hazard ratio for ACM. Intake levels below or above were linked to increased risk, which supports the necessity of balanced consumption of dietary flavonoids.

**Fig.4:**
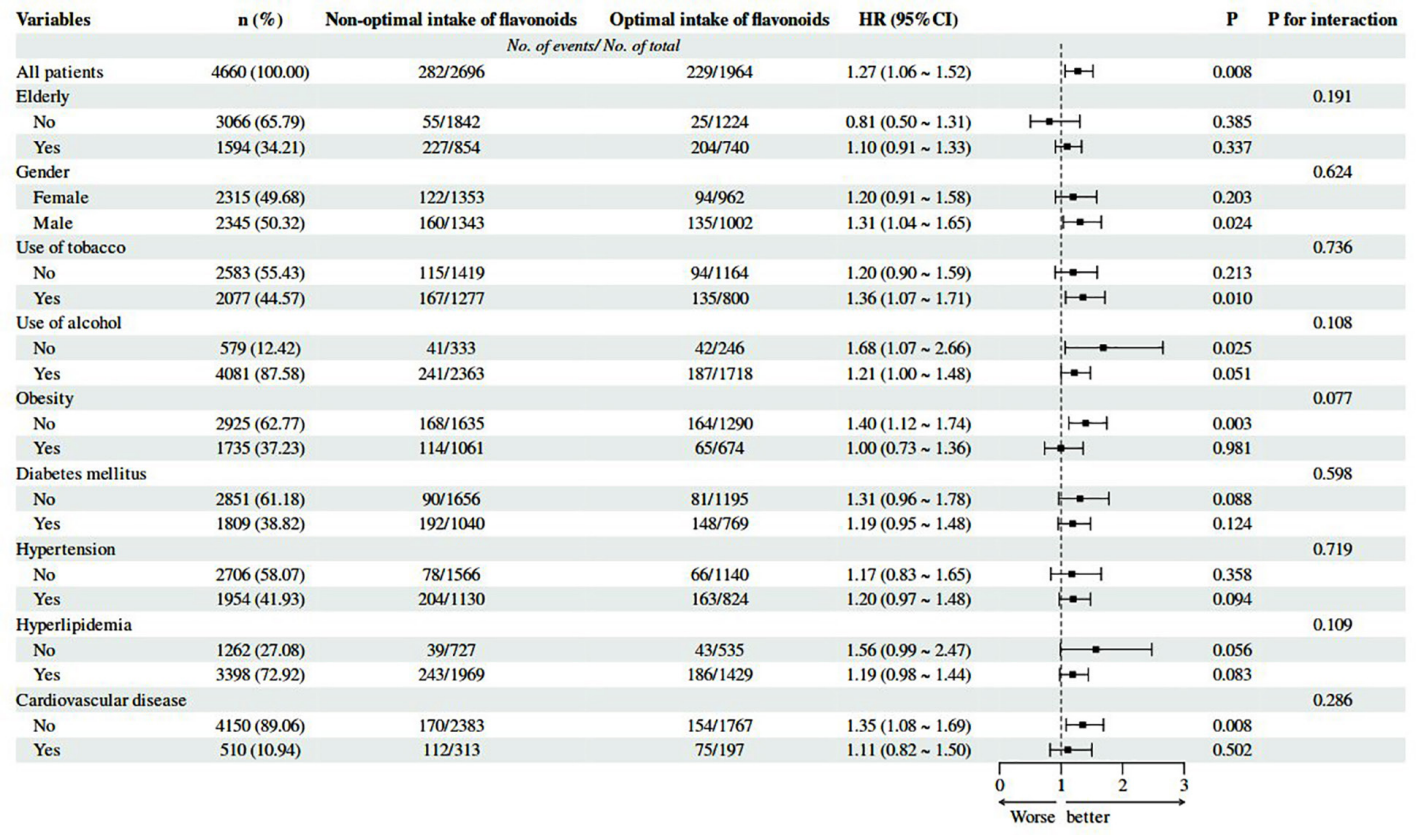
presents a subgroup analysis and a forest plot illustrating the association between dietary flavonoid intake and al-cause mortality.

Subgroup analysis (Fig.4) showed that a few demographic groups got more benefits from optimal flavonoid intake. Specifically, males (P = 0.024), smokers (P = 0.010), non-drinkers (P = 0.025), non-obese individuals (P = 0.003), and those without cardiovascular disease (P = 0.008) exhibited reduced ACM when their flavonoid intake fell within the optimal range. This implies that flavonoid consumption might offer benefits to these specific populations, potentially due to variations in baseline oxidative stress levels or other metabolic factors.

## DISCUSSION

This study examined the link between dietary flavonoid consumption and ACM among a large, nationally representative sample of U.S. adults. The results indicate a U-shaped correlation between flavonoid consumption and ACM, suggesting that both extremely low and extremely high levels of flavonoid intake may elevate the risk of mortality. Comparatively, moderate consumption is linked to a lower risk. This U-shaped pattern matches previous research showing that moderate flavonoid intake is beneficial, but excessive intake may cause adverse effects due to potential pro-oxidative activity.^1^,^5^

In this study, the optimal flavonoid intake range was 48.93 to 735.65 mg/day, which supports earlier research that moderate flavonoid consumption is protective against cardiovascular disease and other chronic conditions.^3^,^4^ Flavonoids have antioxidant and anti-inflammatory properties, which might help reduce oxidative stress and systemic inflammation, both linked to lower risks of chronic diseases and mortality.^6^,^11^ Research consistently shows that diets with high flavonoid content significantly improve endothelial function, optimize lipid metabolism, and effectively regulate blood pressure, all of which are key determinants for maintaining optimal cardiovascular health.^7^,^8^

The subgroup analysis of this study showed that male participants, current smokers, non-alcoholic consumers, individuals with normal BMI (non-obese), and those without a history of cardiovascular disease benefited the most from moderate intake of flavonoids. These findings are consistent with the research results of Hertog et al.^12^ and Arts et al.,^13^ indicating that the effects of flavonoids may vary depending on gender and other demographic factors. For example, men and smokers may experience higher levels of oxidative stress, which can be counteracted by the antioxidant properties of flavonoids.^12^-^15^ Additionally, non-drinkers and non-obese individuals may also benefit from these effects, as their baseline levels of oxidative damage are typically lower.^16^,^17^

Previous studies have showed that flavonoids have a protective effect on cardiovascular diseases by improving glucose metabolism (reflected by reducing fasting blood glucose levels and lowering HbA1c), better controlling systolic blood pressure, etc.,^10^,^18^ due to antioxidant, anti-inflammatory, and vasodilatory properties of flavonoids. Therefore, consuming foods rich in flavonoids may be beneficial as an adjuvant to traditional methods for preventing cardiovascular disease.^19^,^20^ Our findings further support the cardioprotective effects of flavonoids by demonstrating inverse associations with key cardiovascular risk factors. These combined effects suggest that dietary intake of flavonoids may be an important factor in preventing cardiovascular disease and reducing overall mortality.^20^ Furthermore, recent studies from 2022 to 2025 have advanced our understanding of flavonoid metabolism and exposure through metabolomics approaches and urinary biomarkers. For example, urinary flavonoid metabolite profiles identified in humans have been shown to correlate with estimated dietary flavonoid intake, validating their use as objective exposure biomarkers.^21^ In addition, epidemiological and mechanistic studies indicate that flavonoids and their metabolites may exert systemic effects modulating oxidative stress, inflammation, gene expression, and particularly gut microbiota composition processes that have been linked to cardiometabolic health and reduced mortality risk.^22^ Incorporating such biomarker and mechanistic evidence strengthens the biological plausibility of the associations observed in our study.

However, the observed U-shaped association raises questions about potential adverse effects at higher intake levels. While flavonoids are generally recognized for their protective effects, excessive intake particularly through supplements or high-dose extracts may induce pro-oxidative activity under certain conditions. High concentrations of flavonoid metabolites have been shown to generate reactive oxygen species (ROS) or disrupt redox homeostasis.^23^ In addition, flavonoids may interfere with hepatic drug-metabolizing enzymes, such as cytochrome P450 isoforms, potentially altering the metabolism of co-administered medications a concern particularly relevant for older adults or those on polypharmacy regimens.^24^ Moreover, excessive polyphenol intake may result in nutrient–nutrient interactions, such as impaired absorption of essential minerals like iron and zinc due to chelation effects.^25^ These biological mechanisms provide plausible explanations for the increased mortality risk observed at the upper end of flavonoid intake and underscore the importance of identifying an optimal intake range.

Notably, while prior studies have explored associations between flavonoid intake and mortality, our study differs in key aspects.^2^,^4^ First, we used data from a racially and socioeconomically diverse U.S. population, which enhances generalizability.^10^ Second, we applied a refined modeling technique (restricted cubic splines) to identify a specific intake range associated with the lowest risk, which has been less commonly evaluated in previous epidemiologic studies.^1^,^3^ Third, our analysis included stratified subgroup evaluation to reveal differential benefits, which were rarely examined in earlier research.^1^

To enhance the translational value of our findings, we provide approximate dietary equivalents for the optimal flavonoid intake range (48.93–735.65 mg/day). For example, 100 mL of brewed green tea contains approximately 80–120 mg of flavonoids,^8^ while a typical serving of berries, citrus fruits, or apples provides 50–100 mg.^15^ Therefore, a daily intake of 2–3 servings of fruit and 1–2 cups of tea would generally fall within the beneficial intake range identified in this study. Moreover, current dietary guidelines, such as those from the USDA and WHO, do not provide specific recommendations on daily flavonoid or polyphenol intake.^8^,^19^ Our findings may help inform future evidence-based revisions to dietary guidelines, public health strategies, and personalized nutrition messaging by offering data-driven benchmarks.

### Limitations:

First, dietary flavonoid intake was assessed using a single 24 hours dietary recall, which may be subject to recall bias and day-to-day variation, and may not fully reflect habitual intake. Second, we used a complete-case analysis approach, excluding participants with missing data across dietary, biochemical, and clinical domains. While this method is common in NHANES-based studies, it may introduce selection bias if excluded individuals differ systematically. However, most missingness in NHANES is due to its modular survey design rather than participant attrition, and we adjusted for a wide range of covariates to mitigate bias. Third, although our models included numerous covariates to minimize confounding, total energy intake and dietary quality indices were not included, and flavonoid intake was not energy-adjusted. These omissions may lead to residual confounding. Fourth, while subgroup analyses were conducted based on pre-specified variables, additional effect modifiers such as age, race/ethnicity, and comorbidity burden were not explored due to limited statistical power. Fifth, we did not perform further sensitivity analyses such as excluding early deaths or applying penalized spline regression. These could have strengthened the robustness of the observed U-shaped association and are recommended for future research. Sixth, extensive covariate adjustment may raise concerns about overfitting and overadjustment. Some variables, such as hypertension and systolic blood pressure, may also share overlapping clinical meanings. Nevertheless, all variables were selected based on biological relevance and prior literature, and multicollinearity was assessed using Variance Inflation Factors (VIFs), with no significant issues identified. Finally, as this is an observational study, causal inference cannot be drawn. Further randomized controlled trials are needed to confirm these associations and guide evidence-based dietary recommendations.

## CONCLUSION

This study shows that people who consume moderate amounts of dietary flavonoids have a lower risk of all-cause mortality. The study identified specific populations, including males, smokers, and individuals without a history of cardiovascular disease, who would benefit the most from flavonoid supplementation. These findings show that personalized dietary recommendations may be important to optimize flavonoid intake and enhance health outcomes. However, further research is required to uncover the underlying mechanisms and pinpoint the specific flavonoid subclasses that provide the most substantial health benefits.

### Authors’ contributions:

**JW:** Study design, literature search and manuscript writing.

**JW and YW:** Data collection, data analysis and interpretation. Critical review.

**JW:** Manuscript revision and validation and is responsible for the integrity of the study.

All authors have read and approved the final manuscript.
